# Pattern formation in Faraday instability—experimental validation of theoretical models

**DOI:** 10.1098/rsta.2022.0081

**Published:** 2023-04-17

**Authors:** B. Dinesh, J. Livesay, I. B. Ignatius, R. Narayanan

**Affiliations:** ^1^ Department of Chemical Engineering and Technology, Indian Institute of Technology-BHU, Varanasi, 221005, Uttar Pradesh, India; ^2^ Department of Chemical Engineering, University of Florida, Gainesville, FL 32611, USA

**Keywords:** Faraday instability, electrostatic Faraday, pattern formation

## Abstract

Two types of resonance-derived interfacial instability are reviewed with a focus on recent work detailing the effect of side walls on interfacial mode discretization. The first type of resonance is the mechanical Faraday instability, and the second is electrostatic Faraday instability. Both types of resonance are discussed for the case of single-frequency forcing. In the case of mechanical Faraday instability, inviscid theory can forecast the modal forms that one might expect when viscosity is taken into account. Experiments show very favourable validation with theory for both modal forms and onset conditions. Lowering of gravity is predicted to shift smaller wavelengths or choppier modes to lower frequencies. This is also validated by experiments. Electrostatic resonant instability is shown to lead to a pillaring mode that occurs at low wavenumbers, which is akin to Rayleigh Taylor instability. As in the case of mechanical resonance, experiments show favourable validation with theoretical predictions of patterns. A stark difference between the two forms of resonance is the observation of a gradual rise in the negative detuning instability in the case of mechanical Faraday and a very sharp one in the case of electrostatic resonance.

This article is part of the theme issue ‘New trends in pattern formation and nonlinear dynamics of extended systems’.

## 1. Introduction to the theme of this article

An instability may arise when two immiscible liquids are subject to periodic forcing normal to their common interface. This instability manifests itself in the form of surface undulations and is due to the resonance that can occur when the parametric or enforced frequency of oscillations is commensurate with the system’s natural frequency. In this article, we review the main works of the last decade on resonant instability at fluid interfaces in the context of modal shapes that appear at the surface, first due to mechanically induced oscillations and then due to electrostatically induced oscillations. Comparisons of the theory with experiments is a main characteristic of this review.

## 2. Mechanical Faraday instability

### (a) Introduction

**Figure 1.**
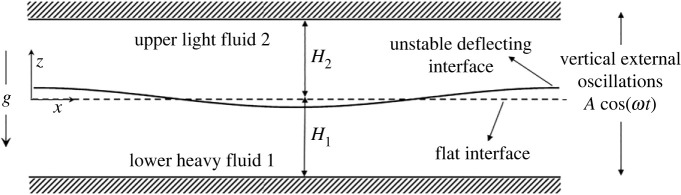
Schematic depicting two layers, of heights $H_{1}$ and $H_{2}$, subject to vertical oscillations as done in a Faraday experiment. The amplitude of shaking is A, and the frequency is ω. The upper fluid is denoted as fluid 2, and the bottom fluid is denoted as fluid 1.

When a mechanically oscillating acceleration field is applied perpendicular to the common interface of two fluids, an instability, manifested by the sudden generation of waves and fluid motion at the interface, occurs and is termed the Faraday instability [1]. Figure 1 depicts a periodically forced bilayer, light fluid overlying a heavy fluid, moving with a forcing frequency of ω, and amplitude, A, yielding a forcing acceleration field of $A\omega^{2}$.^1^ The view to be taken is that the bilayer is shaken at a set frequency, ω, and the amplitude of the motion, A, is incrementally changed until the instability occurs. *The instability is a result of resonance between the imposed or parametric frequency of oscillation and the system’s natural frequency*. Now, the natural frequency and, therefore, the threshold amplitude are affected by gravity and surface tension among other factors.

A bilayer’s natural frequency, $\omega_{N}$ (not to be confused with ω) is the frequency of oscillations that the layers experience when an initial pulse disturbance is given to an erstwhile quiescent state. Of course, both the velocity field and the interface decay due to the viscosities of the layers. This decay is non-monotonic, and the oscillation frequency experienced by each modal pattern during the decay is the specific mode’s natural frequency. In general, the natural frequency must be computed numerically. The natural frequency depends upon the density difference of the fluid layers multiplied by gravity, the interfacial tension, the viscosities, the layer heights and the geometry or shape of the mode. These modal shapes often termed *discretized modes or shapes* in this article are dictated by the lateral geometry of the container. For example, if the side walls are slippery, these shapes would be trignometric in the case of rectangular geometries and cylindrical harmonics, i.e. obtained from the roots of the derivatives of the Bessel’s functions in the case of circular cross-section, and so forth.

Unlike the case of viscous bilayers, an analytical expression for the natural frequency can be easily determined if the fluids are imagined to be inviscid. One accomplishes this by considering the inverse time constant of the system’s response, σ, to a disturbance of infinitesimal amplitude about the quiescent state. This is done by expressing the velocity perturbations in the form $v=e^{\sigma t}v{\;}^{\prime }$ and likewise for pressure and interface perturbations. Then σ is determined from a resulting eigenvalue problem for the disturbance or primed variables. For example if the side walls are taken to be periodic, then expansion of the velocity, pressure and interface perturbations in horizontal modes of the form $e^{ikx}$, with disturbance wavenumber, k, yields an expression for $\sigma^{2}$ whence the frequency, $\omega_{N}=Im(\sigma )$, is obtained. This expression is

**Figure 2.**
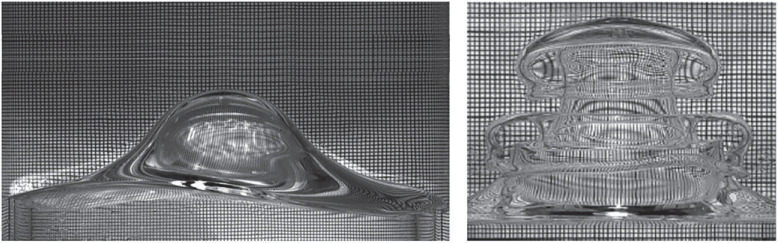
Photographs of discretized standing wave and a wave at breakup slightly post-onset of the instability in a circular cylindrical geometry from [4]. The breakup wave is termed subcritical instability, while the standing wave is supercritical. Reprinted with permission from Cambridge University Press.

2.1 $$\sigma^{2}=-\frac{k(g\Delta \rho +\gamma k^{2})}{\left(\rho_{1}+\rho_{2})\coth(kh\right)},$$

where h is the depth of the fluid layers (i.e. $H_{1}$ and $H_{2}$ in the figure are taken to be equal to one another for simplicity and replaced here by h). The variables on the right-hand side of equation (2.1) are Δρ, the positive density difference; g, the gravitational constant; γ, the interfacial or surface tension; and k, the disturbance wavenumber. It must be noted that if the fluid layer is of infinite lateral extent, the wavenumber, k, is a continuous variable yielding a continuous spectrum of natural frequencies. Laterally bounded fluids lead to discretized modes or combination of modes depending on the side wall conditions. It is seen from equation (2.1) that σ is purely imaginary as the fluids are taken to be inviscid in obtaining the aforementioned expression. In a viscous bilayer, however, σ would have a negative real part, indicating decay and an imaginary part that determines the frequency of the decaying mode. When the forcing frequency is commensurate with the natural frequency of a mode, resonance occurs and the instability with that modal wave form appears. For large forcing frequencies, the effect of the side walls are not felt, and the resulting waves are of small wavelength compared to the side wall spacing. But when the forcing frequencies are small, the side wall geometry plays a strong role in the wave shape and the modes are discretized. A typical discretized modal wave form at the onset of instability may be seen in the photographs of figure 2. Several comments on equation (2.1) and figure 2 are in order. It is apparent from equation (2.1) that large forcing or parametric frequencies, ω, lead to resonance when the wavenumber increases, i.e. wavelengths become small. When viscosity is also taken into account, short wavelengths are stabilizing due to viscosity in addition to surface tension, leading us to think that the threshold value of the forcing amplitude, A, ought to increase. However, acceleration is given by $A\omega^{2}$, therefore leading us to think that the amplitude ought to fall. There is thus a trade-off in stability with respect to ω. Theoretical models tell us that for a given modal pattern, the * threshold amplitude* decreases with ω, reaches a minimum and then increases, reflecting this trade-off [5]. This is also seen later in figure 7.

**Table 1.** Fluid properties for FC-70 and silicone oil.

physical properties	FC-70	silicone oil
density ρ (${\mathrm{kg}\;m}^{-3}$)	1916	846
kinematic viscosity ν ($m^{2}\;s^{-1}$)	$10\times 10^{-6}$	$1.5\times 10^{-6}$
fluid thicknesses (mm) $H_{1},H_{2}$	21	20
interfacial tension γ (${N\;m}^{-1}$)	0.007	

A second set of observations arise from figure 2. This figure depicts two types of typical Faraday modal wave forms seen immediately after the onset of the instability, the main difference being the parametric frequency at which the instability occurs. In one, a standing wave pattern is observed, while in the other, the interface is seen to break up. The first is termed supercritical instability meaning that waves can saturate to time-periodic behaviour after the onset of the instability. The second is termed subcritical instability, indicating that the waves *break up catastrophically* immediately after the onset. This breakup is manifested by the fluids exchanging positions. The precise physical reasons for the different behaviors are not well understood even though they have been observed by several workers [4,6,7]. However, what is known from the theory is that the instability is influenced by the base pressure gradient [5]. This gradient is given by $\Delta \rho (-g+A\omega^{2}\;\cos(\omega t))$ of which the first term is stabilizing since the bilayer configuration is light on top of heavy. The second term leads to resonance and is somewhat reminiscent of the Rayleigh–Taylor problem, which is subcritical in nature, since heavy fluid accelerates into the light fluid during one half of the cycle [8–10]. As stated earlier, for a given modal wave pattern of response, the critical forcing amplitude at first decreases with ω, reaches a minimum, and then increases until the next modal pattern is formed. In the decreasing region, the instability is experimentally seen to be subcritical while it is seen to be supercritical in the ascending region [4]. For very large ω, however, all discretized modes disappear, and the waves become very choppy with only saturated stable waves occurring.

For our final set of observations, we return to equation (2.1) which yields a scaled group that compares gravitational effects to interfacial tension effects. It is $\left[g\Delta \rho W^{2}/\gamma \right]$, where W, the width of a container, determines the allowable wavenumbers. Thus, to have resonant instability with negligible gravity effects, either g must be made small or W must be made small, i.e. small width containers must be used. Note that density matching is of no use in reducing the effect of g, because a density matched bilayer will simply oscillate as a rigid body and *resonance would then not be possible*. This is also apparent because the net base pressure difference at the interface, i.e. $\Delta \rho [-g+A\omega^{2}\;\cos(\omega t)]$, is zero when Δρ is zero. In fact, the smaller the density difference, the greater the required amplitude, A, for the onset of the instability, all other variables being held fixed. Likewise the smaller the width of a container, the greater the sidewall damping and the greater the forcing amplitude. More observations can be made from equation (2.1). From the equation, we see that γ, the interfacial or surface tension, is multiplied by $k^{2}$. This means that, if g were eliminated, we might expect that the onset of the instability in a wide container will be manifested by choppy waves, i.e. waves with large wavenumber, k. In other words, we ought to see a shift toward smaller wavelength, *yet discrete modes in experiments at low g compared to identical experiments under Earth’s gravity.* We now turn to a theoretical model whose predictions are shown to be validated by experiments.

### (b) Theoretical model

The theoretical model determines the thresholds and the behaviour of the flow near the instability. We first linearize the nonlinear equations about the base state and then inspect the eigenvalues of the resulting homogeneous problem [11]. The base state is quiescent with a flat interface with respect to an observer, situated on the moving oscillating frame.

#### (i) Governing equations

To obtain the non-dimensional governing equations, characteristic scales are needed where the subscript i refers to the fluid phase, the index, 1, refers to the lower phase and 2 to the upper phase. The characteristic length, time, velocity and pressure are then given by,

2.2 $$l_{c}=H_{1},\;t_{c}=\sqrt{\frac{\rho_{1}H_{1}^{3}}{\gamma }},\;U_{c}=\frac{H_{1}}{t_{c}}=\sqrt{\frac{\gamma }{\rho_{1}\;H_{1}}},\;p_{i,c}=\rho_{1}\;U_{c}^{2}.$$

In a frame of reference moving along with the fluid-carrying container, the governing equations are as follows:

2.3 $$\nabla \cdot {\text{v}}_{i}=0$$

and

2.4 $${\left(\rho \right)}^{\delta_{i2}}Re\left(\frac{\partial {\text{v}}_{i}}{\partial t}+{\text{v}}_{i}\cdot \nabla {\text{v}}_{i}\right)=-Re\;\nabla p_{i}+{\left(\mu \right)}^{\delta_{i2}}\nabla^{2}{\text{v}}_{i}-{\left(\rho \right)}^{\delta_{i2}}(G+A\cos(\Omega t))\;{\text{e}}_{z}.$$

Here, $\rho =\rho_{2}/\rho_{1}$, $\mu =\mu_{2}/\mu_{1}$ $Re=\rho_{1}U_{c}H_{1}/\mu_{1}$, $G=\rho_{1}gH_{1}^{2}/\mu_{1}U_{c}$, $A=\frac{\rho_{1}A\omega^{2}H_{1}^{2}}{\mu_{1}U_{c}}$, $\Omega =\omega t_{c}$ and $\delta_{ij}$ is the Kronecker delta. In addition to the domain equations given earlier, the conditions at the interface, z=ζ(r,t), are given as follows:

2.5 $$\begin{array}{rl} & \text{n}\cdot {\text{T}}_{1}\cdot \text{t}=\mu \;(\text{n}\cdot {\text{T}}_{2}\cdot \text{t}),\;\text{where},\;{\text{T}}_{i}=\nabla {\text{v}}_{i}+\nabla {\text{v}}_{i}^{T},\;\text{n}=\frac{-(\partial \zeta /\partial x){\text{e}}_{x}+{\text{e}}_{z}}{{\left[1+{\left(\partial \zeta /\partial x\right)}^{2}\right]}^{1/2}},\\ & \text{t}=\frac{{\text{e}}_{x}+(\partial \zeta /\partial x){\text{e}}_{z}}{{\left[1+{\left(\partial \zeta /\partial x\right)}^{2}\right]}^{1/2}}\left(-p_{2}+\frac{\mu }{Re}\text{n}\cdot {\text{T}}_{2}\cdot \text{n}\right)+p_{1}-\frac{1}{Re}\text{n}\cdot {\text{T}}_{1}\cdot \text{n}=\frac{1}{Re\;Ca}\kappa ,\\ & \;\text{where}\;Ca=\frac{\mu_{1}\;U_{c}}{\gamma },\;\kappa =\nabla \cdot \text{n}({\text{v}}_{1}-{\text{v}}_{2})\cdot \text{n}=0,\;({\text{v}}_{1}-{\text{v}}_{2})\cdot \text{t}=0,\;{\text{v}}_{i}.\text{n}-U=0, \end{array}$$

2.7 $$\begin{array}{rl} & \;\text{where the interface speed, }\;U=\frac{\partial \zeta /\partial t}{{\left[1+{\left(\partial \zeta /\partial x\right)}^{2}\right]}^{1/2}}. \end{array}$$

#### (ii) Base state and linear stability

The stability of the base state, i.e. a quiescent state with a flat interface, is analyzed using linear stability theory. All variables (ϕ) are expressed as a sum of the base state ($\bar{\phi }$) and the corresponding perturbations ($\phi^{\prime }$), i.e. $\phi =\bar{\phi }+\epsilon \phi^{\prime }$, where ϵ is a small parameter. At leading order, the base state satisfies:

2.8 $${\bar{\text{v}}}_{i}=0,\;\frac{\partial {\bar{p}}_{i}}{\partial z}=-\frac{{\left(\rho \right)}^{\delta_{i2}}}{Re}(G+A\cos(\Omega t))\;\text{and}\;\bar{\text{n}}={\text{e}}_{z},\;\bar{\text{t}}={\text{e}}_{x}.$$

At O(ϵ), the perturbed variables are governed by:

2.9 $$\begin{array}{rl} & \nabla \cdot {\text{v}}_{i}^{\prime }=0,\;\text{and}\;{\left(\rho \right)}^{\delta_{i2}}Re\frac{\partial {\text{v}}_{i}^{\prime }}{\partial t}=-Re\nabla p_{i}^{\prime }+{\left(\mu \right)}^{\delta_{i2}}\nabla^{2}{\text{v}}_{i}^{\prime }, \end{array}$$

2.10 $$\begin{array}{rl} & \bar{\text{n}}\cdot {\text{T}}_{1}^{\prime }\cdot \bar{\text{t}}=\mu \;(\bar{\text{n}}\cdot {\text{T}}_{2}^{\prime }\cdot \bar{\text{t}}),\\ & \left(-p_{2}^{\prime }+\frac{\mu }{Re}\bar{\text{n}}\cdot {\text{T}}_{2}^{\prime }\cdot \bar{\text{n}}\right)+p_{1}^{\prime }+\left(\frac{\partial {\bar{p}}_{1}}{\partial z}-\frac{\partial {\bar{p}}_{2}}{\partial z}\right)\zeta^{\prime }\\ & \;\;-\frac{1}{Re}\bar{\text{n}}\cdot {\text{T}}_{1}^{\prime }\cdot \bar{\text{n}}=\frac{1}{Re\;Ca}\nabla_{H}^{2}\zeta^{\prime },\;\text{which gives:}\\ & \left(-p_{2}^{\prime }+\frac{\mu }{Re}\bar{\text{n}}\cdot {\text{T}}_{2}^{\prime }\cdot \bar{\text{n}}\right)+p_{1}^{\prime }+\frac{1}{Re}[(\rho -1)G+(\rho -1)A\cos(\Omega t)]\zeta^{\prime }\\ & \;\;-\bar{\text{n}}\cdot {\text{T}}_{1}^{\prime }\cdot \bar{\text{n}}=\frac{1}{Re\;Ca}\nabla_{H}^{2}\zeta^{\prime } \end{array}$$

2.13 $$\begin{array}{rl} \mathrm{and}\; & \;({\text{v}}_{1}^{\prime }-{\text{v}}_{2}^{\prime })\cdot \bar{\text{n}}=0,\;({\text{v}}_{1}^{\prime }-{\text{v}}_{2}^{\prime })\cdot \bar{\text{t}}=0,\;{\text{v}}_{i}^{\prime }.\bar{\text{n}}-\frac{\partial \zeta^{\prime }}{\partial t}=0. \end{array}$$

#### (iii) Floquet theory

As the equations governing the perturbation variables ($\phi^{\prime }$) have non-constant coefficients that are periodic in time, Floquet theory is used to determine the stability of the system. The perturbation variables are expanded as follows:

2.14 $$\phi^{\prime }(x,t)=\sum_{n=-\infty }^{\infty }{\hat{\phi }}_{n}(x){\text{e}}^{(\sigma +i(\alpha +n\Omega ))t}.$$

Here, σ is the growth rate, k is the spatial wavenumber and α is either taken to be zero or Ω/2 for harmonic and subharmonic responses, respectively. To obtain conditions for the onset of instability, σ is set to zero. The summation in (2.14) is truncated at a finite value of N, such that −N≤n≤N, and all terms with ${\text{e}}^{im\Omega t}$, where |m|>N, are ignored. We can then obtain the critical forcing amplitude, A, by solving a generalized eigenvalue problem of the form, $\text{L}{\hat{\phi }}_{i}=A\text{B}{\hat{\phi }}_{i}$. This is the key eigenvalue problem from which the critical amplitude over a frequency sweep is obtained. The outputs are the eigenvalues, A. The lowest of these in magnitude is the threshold amplitude for an assigned parametric frequency. In these calculations, the choice of N is increased until sufficient convergence is achieved. The computations of the eigenvalues involve linear partial differential equations that depend only on the spatial direction. The spectral Tchebychev method may be used to do this [12].

#### (iv) Inviscid limit

In the special case of inviscid fluids, on use of the continuity equations in each phase, we obtain

2.15 $$\nabla^{2}{p_{i}}^{\prime }=0,$$

subject to Neumann conditions at the top and bottom plates, and the following conditions at the reference surface z=0: first,

2.16 $$-p_{2}^{\prime }+p_{1}^{\prime }+[(\rho -1)B+(\rho -1)A_{B}\cos(\Omega t)]\zeta^{\prime }=\frac{1}{We}\nabla_{H}^{2}\zeta^{\prime },$$

where $B=\frac{\rho_{1}gH_{1}^{2}}{\gamma }$ and $A_{B}=\frac{\rho_{1}A\Omega^{2}H_{1}^{2}}{\gamma }$, where We the Weber number is given by $We=\rho_{1}U_{c}^{2}H_{1}/\gamma$ and second,

2.17 $$\frac{\partial p_{i}^{\prime }}{\partial z}=-\rho^{\delta_{i,2}}\frac{\partial^{2}\zeta^{\prime }}{\partial t^{2}}.$$

The variables ${p_{i}}^{\prime }$ and $\zeta^{\prime }$ are then expanded in terms of cos(kx), where k=nπ/W, where W is the scaled width in a one-dimensional system, as the side walls are taken to be stress free. This yields the following evolution equation for the interface deflection, $\zeta^{\prime }$, called the Mathieu equation.

2.18 $$\frac{\partial^{2}\zeta^{\prime }}{\partial t^{2}}+[p-2q\;\cos(\Omega t)]\zeta^{\prime }=0.$$

Here, p and q are given by

2.19 $$p=\frac{(1-\rho )\;B\;k+k^{3}}{\left(\coth(k)+\rho \;\coth(k\;H)\right)}\;\text{and}\;q=\frac{(1-\rho )\;A_{B}\;k}{2(\coth(k)+\rho \;\coth(k\;H))}.$$

It is common to re-express equation (2.18) by redefining τ=Ωt/2, $\tilde{p}=4p/\Omega^{2}$ and $\tilde{q}=4q/\Omega^{2}$. Equation (2.18) now becomes the general form of the Mathieu equation:

2.20 $$\frac{\partial^{2}\zeta^{\prime }}{\partial \tau^{2}}+[\tilde{p}-2\tilde{q}\;\cos(2\tau )]\zeta^{\prime }=0.$$

**Figure 3.**
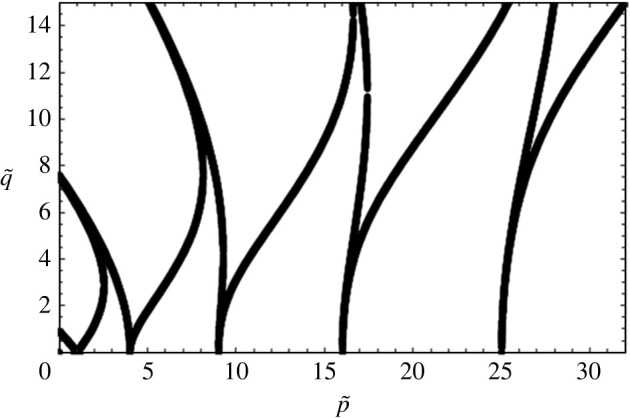
A plot of $\tilde{q}$ versus $\tilde{p}$ obtained upon solving the Mathieu equation (2.18) [13]. The plot is generated using the Floquet theory.

**Figure 4.**
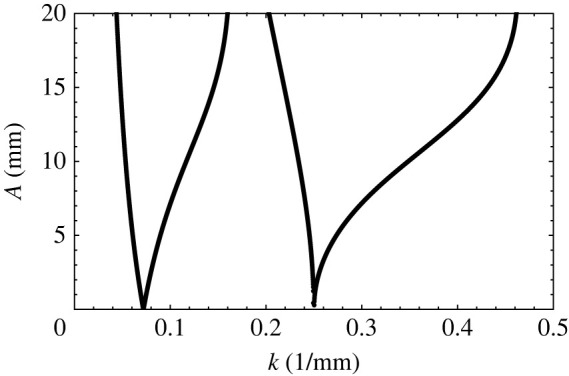
Amplitude (mm) versus wavenumber (1/mm) obtained from the inviscid theory for an applied frequency of 5 Hz. The physical properties of the fluids and the fluid depths are given in table 1. The first tongue is subharmonic, and the second tongue is harmonic.

This equation is solved by expressing $\zeta^{\prime }$ in a series of the form given by equation (2.14). A master curve of $\tilde{p}$ vs $\tilde{q}$ results and is shown in figure 3. Now If we assume fluid properties as shown in table 1, however assuming the fluids to be inviscid, a second curve, figure 4, results. Here, for a given input of Ω, a typical curve of A vs $k^{2}$ is seen. Notice in this figure that various ‘tongues’ appear and that in each tongue, the amplitude A approaches zero at certain values of $k^{2}$. At these points, known as resonant wavenumbers, q=0 since A=0, and from equation (2.14), it follows that each of the modes, n, separate from one another. In other words, $\hat{\zeta_{n}^{\prime }}$ are zero for all but one value of n. At the resonant value of k for the first tongue, we see that the first element of the eigenfunction, i.e. $\hat{\zeta_{0}^{\prime }}$ alone, is non-zero, while all other $\hat{\zeta_{n}^{\prime }}$ are zero. This tongue, therefore, has a subharmonic response and is the tongue corresponding to α=Ω/2. At the resonant point of the second tongue $\hat{\zeta_{1}^{\prime }}\neq 0$, while all other $\hat{\zeta_{n}^{\prime }}$ are zero, corresponding to α=0 with a harmonic response and so forth for all other tongues. As we move to the other tongues, not shown in the figure, we see that the responses will be 3/2 harmonic, twice harmonic and so forth. Another way of viewing the figure is to insert the value of a resonant k (say from the first tongue) into equation (2.1) and observe that the value of $\omega_{n}$ would be precisely equal to the unscaled value of Ω/2. If we insert the value of k at the resonant point of the second tongue into equation (2.1), we ought to obtain Ω and so forth.

While figure 4 is a convenient way to display a calculation from equation (2.18), it is not a practical way to view the problem. To take such a view, it would appear that a graph of A vs frequency in Hz would be of use, for a given container width (or radius). To do this, choose a radius, R, say for a cylindrical geometry, and then obtain k the allowable wavenumbers from the various roots of the Bessel’s functions $J_{m}^{\prime }(k_{n}\;R)=0$, noting that n represents the n th root, while m is the azimuthal mode. Then a plot of A vs frequency could be drawn for the allowable wavenumbers.

**Figure 5.**
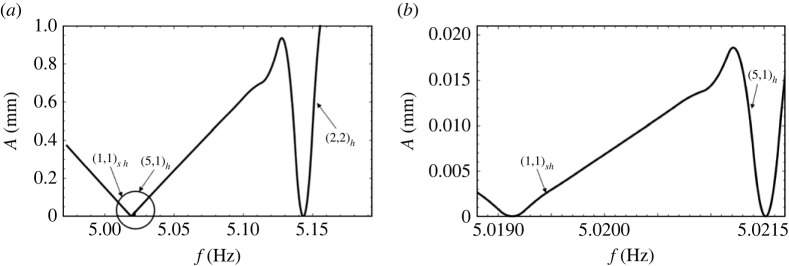
The critical A versus frequency plot for a bilayer of FC70 and silicone oil ignoring viscosities. Note that the amplitude of forcing is zero for the frequencies: (*a*) 5.019 Hz and 5.143 Hz and (*b*) 5.019 Hz and 5.021 Hz. The physical properties of the fluids and the fluid depths are given in table 1. The radius of the Faraday cell is 0.025 m.

We can get some assistance by calculating the natural frequencies for a set of allowable wavenumbers from equation (2.1). At those values of the natural frequencies, A should be zero, and the response must be harmonic. Table 2 is a display of such frequencies in a range between 3 Hz and 7 Hz. Now, if we were to take as an example an assigned smaller sub-range, of say, between 4.75 Hz and 5.25 Hz, we would arrive at just two natural frequencies, i.e. 5.021 and 5.14. At these frequencies, A is zero with a harmonic response. A plot of A vs frequency, f, is shown in figure 5*a*. This figure is somewhat deceiving as a blowup of the region near the left point shown in figure 5*b* shows two tongues near the lower frequency of figure 5*a*. To see why this is so, we search the range between 4.75/2 and 5.25/2, i.e. the range that is precisely half of the original range, and we find a natural frequency of 2.509 Hz with an accompanying mode of (1,1). This means that a subharmonic response of twice 2.509 Hz will occur in our assigned range, i.e. at 5.019 Hz. This is why the blowup of figure 5*a* depicts an extra tongue at 5.019 Hz with mode (1,1). This observation does not preclude the occurrence of yet higher harmonics, i.e. 3/2,2,5/2, etc., harmonics inside our assigned range. However, for simplicity, we should focus only on the first three responses, two harmonics and one subharmonic. Notice that the harmonic responses have modal representations of (5,1) and (2,2), while the subharmonic response has mode of (1,1). This makes the subharmonic mode the least ‘choppy’ of the modes in our assigned range of interest. If viscosity is added to the model it might be conjectured that A cannot ever become zero and that the choppier of the modes would have a higher critical value of A making the least choppy mode in the set, i.e. (1,1) to be the dominant mode near the forcing frequency. We can see this when looking at figure 7 where now the (1,1) mode is what is seen in the vicinity of 5 Hz. This takes us to a discussion of viscous effects.

**Table 2.** Natural frequencies and the corresponding modes obtained from equation (2.1).

natural frequencies	3.822	2.509	3.386	4.011	4.541	5.021
modes	(0,1)	(1,1)	(2,1)	(3,1)	(4,1)	(5,1)
natural frequencies	5.271	4.548	5.143	5.676	6.172	6.646
modes	(0,2)	(1,2)	(2,2)	(3,2)	(4,2)	(5,2)

#### (v) Viscous effects and experimental validation

**Figure 6.**
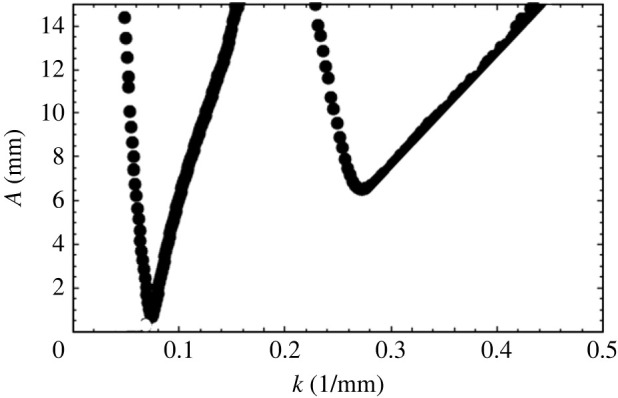
Amplitude (mm) versus wavenumber (1/mm) obtained from viscous theory for an applied frequency of 5 Hz. The physical properties of the fluids and the fluid depths are given in table 1. Note that the Faraday tongues do not touch the x-axis. The first tongue is subharmonic, and the second tongue is harmonic.

**Figure 7.**
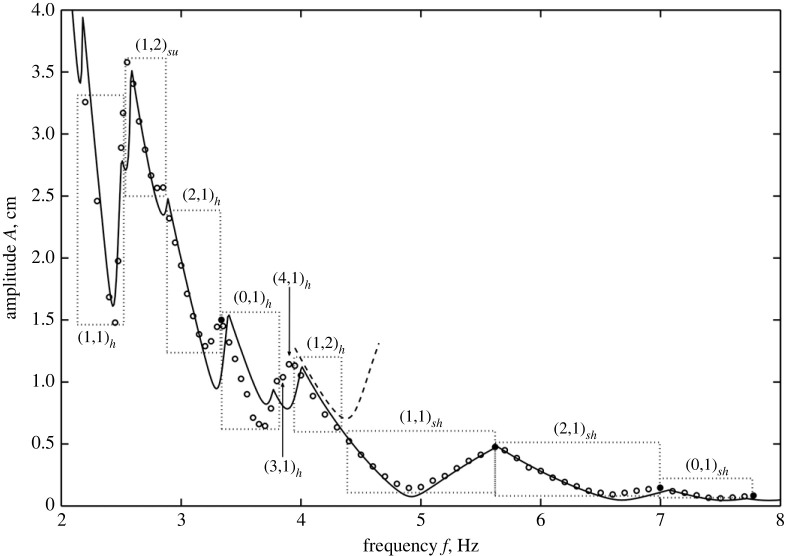
Comparison of amplitude (cm) versus frequency (Hz) obtained from theory (solid curve) and the experiments (open circles while filled circles represent co-dimension 2 points). The physical properties of the fluids and the fluid depths are given in table 1. The radius of the Faraday cell is 0.025 m [4]. Reprinted with permission from Cambridge University Press.

In a major re-working of the Benjamin and Ursell theory, now taking viscosity into account, Kumar and Tuckerman [14] did a calculation that depicted A versus k (compared with figure 6) showing an increase in the minimum A for each harmonic tongue compared with the counterpart inviscid case. The increase in A for increasing harmonics is a result of viscous damping for increasingly ‘choppy’ modes. The corresponding A vs f curve is depicted in figure 7 where now experimental data marked in open circles are also shown.

This figure shows a comparison between experiment and theoretical prediction of the case of a circular cylindrical geometry. The experiment used two fluids, 1.5 centistokes silicone oil over a flourinated oil named FC 70 of viscosity 12 centistokes. An interesting feature of this two-fluid combination placed in a glass cylinder is the coating offered by the upper fluid along the side walls, thereby closely mimicking stress-free conditions. Thus if the diameter of the cylinder is several times the depth of the fluids, one can expect that the damping arising from mensicus waves will be minimized, yet discrete modes can appear at the onset of the instability. This is what the figure also depicts.

**Figure 8.**
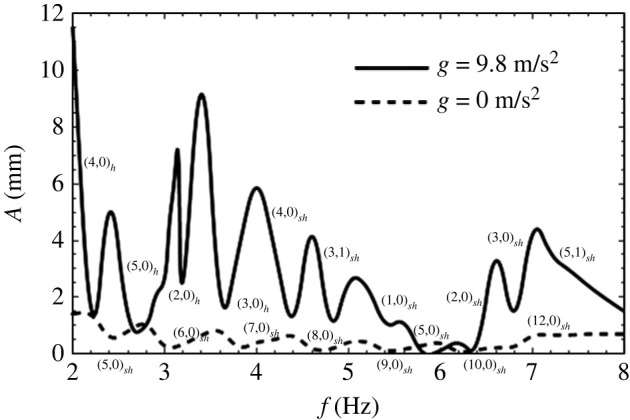
Amplitude (mm) versus frequency (Hz) for g=0 and g=9.8 in a rectangular geometry. The physical properties, fluid depths and cell dimensions are given in table 3 (compared with [21]). The first index is the number of half waves in the long direction, and the second index is the number of half waves in the short direction.

**Figure 9.**
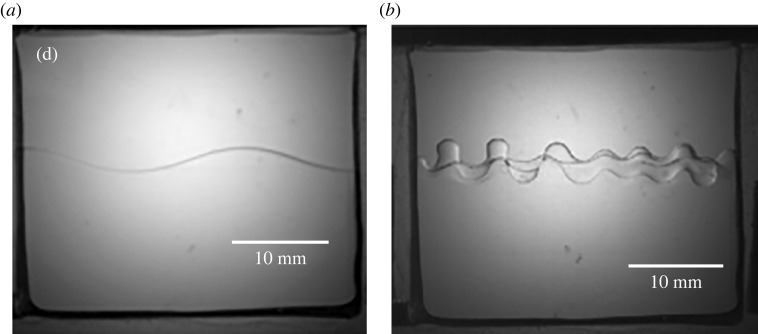
The waveforms obtained at the interface for (*a*) $g=9.8\;{m\;s}^{-2}$ and (*b*) microgravity environments for forcing frequency of about 7 Hz each. Observe the choppiness in the wave structure at microgravity. There are three half waves under 1 g and 14 half waves under microgravity [21]. The physical properties, fluid depths and cell dimensions are given in table 3 (compared with [21]). Reprinted with permission of the publisher.

There are other comments on the figure that may be made. First, as expected from the inviscid case, the curve is not monotonic depicting minima in the thresholds for various modes. The lowest or minimum point in each modal range is very close to the system’s natural frequency and coincides with it only in the limit of vanishing viscosity. This implies that the Faraday forcing method is a plausible way to experimentally estimate the natural frequencies provided the Reynolds number is high. Of course, as we saw earlier, more modes creep in the vicinity when the effect of viscosity is reduced. As a case in point near the 5 Hz location, we have three modes in the inviscid case, i.e. (1,1), (5,1) and (2,2), but only one survives, i.e. (1,1), when viscosity is added, this being the least choppy one. In fact, near the frequency of 4.5 Hz, a choppier but harmonic mode, (1,2) that is slightly more stable is depicted and is forecast from table 2. The closeness of the modes makes it difficult to discern the behaviour when modes are close to one another. A second observation is that the peaks of the curve represent the transition of one modal pattern to another and are termed co-dimension two points, represented by filled circles. These represent points where two eigenforms coincide for the same eigenvalue A. Third, the descending parts of the curve, i.e. the parts with negative slope, represent the positive detuning towards the natural frequency and are experimentally seen to manifest as subcritical instability. This means that the interface goes towards rupture along these parts of the curve. The regions of the positive slope are the negative detuning parts and are seen experimentally to manifest as saturated steady waves. The precise reason for this behaviour is a matter of current inquiry. Fourth, the agreement between theoretical predictions and experiment is startling, with the exception of one region demarked (0,1). The experimental points appear well below the theoretical predictions here. This is attributed to the mensicus waves that are axisymmetric and harmonically interfere with the response, causing an ‘imperfect bifurcation’ [15]. The remarkable agreement between the theory and the experiment is partly due to the choice of fluids where the meniscus waves play a minimal role due to the distance of the side walls and also due to the periodicity of the wave forms in the angular direction. These advantages are lost if a rectangular geometry is chosen, and the side walls are of close proximity [16]. There are several studies of mechanical Faraday experiments in close bounded geometries that show the side wall effects. These have been reviewed extensively by Batson *et al.* [4] and compared with previous studies [17–19]. A recent article by Wilson *et al.* very nicely and interestingly shows that the addition of a suitable surfactant such as Tritton in the aqueous solution has the effect of reducing side wall non-ideality [20]. As we exit this section, we comment on the effect of gravity on the response to Faraday forcing. Again, the forcing is in the direction normal to the erstwhile flat surface unlike the case when the shaking is parallel to the surface [3]. Calculations done for a rectangular geometry, compared with figure 8, show that there is a definite shift in the modes as we decrease gravity. In general, we can say that the responding waveforms become more choppy in microgravity. This result is depicted in experiments and displayed in figure 8, while a typical experiment done in microgravity as compared with one done under Earth’s gravity is displayed in figure 9 (see table 3 for the physical properties of the fluids). We observe that under the same forcing frequency the microgravity wave lengths are definitely much smaller as predicted by equation (2.1). The modal forms in the experiments depicted in figure 9 are in close agreement with the theoretical prediction depicted in figure 8. The available modes are subharmonic, an observation that has not been explained in physical terms.

**Table 3.** Fluid properties for FC-72 and silicone oil.

physical properties	FC-72	silicone oil
density ρ (${\mathrm{kg}\;m}^{-3}$)	1680	816
kinematic viscosity ν ($m^{2}\;s^{-1}$)	$0.38\times 10^{-6}$	$1.0\times 10^{-6}$
interfacial tension γ (${N\;m}^{-1}$)	$0.77\times 10^{-3}$	
width of Faraday cell (m)	$35\times 10^{-3}$	
depth of Faraday cell (m)	$5\times 10^{-3}$	
heights of the fluids (m)	$14.65\times 10^{-3}$	$14.65\times 10^{-3}$

## 3. Electrostatic resonance

### (a) Introduction

**Figure 10.**
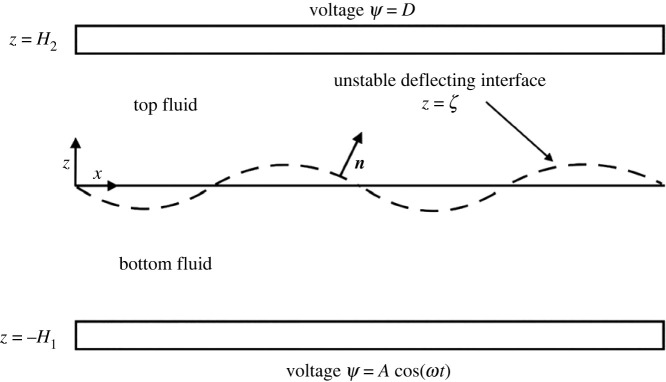
Schematic of electrostatic instability. Here a lighter fluid (silicone oil or fluid 2) lies on top of a heavy fluid (water or fluid 1). An oscillatory potential field, i.e. A cos(ω t), is applied at the bottom plate and a constant DC field, D, is applied at the top plate. Here, water is taken to be a perfect conductor and silicone oil is taken to be a perfect dielectric.

In the earlier section, our focus was on resonance via mechanical forcing. We observed that the effect of surface tension could be enhanced by reducing gravity. However, reducing gravity by going to outer space is not the only way to bring out the effect of surface tension. Another way requires the use of a countering force. One way to achieve this is by introducing a DC electrostatic field on the bilayer of fluids provided the fluids have appropriate electrical properties. As an example, take the fluids to be a conducting/dielectric pair with a DC field imposed on the system as depicted in figure 10. If the interface were to be perturbed in the presence of the DC field, it would restore to its equilibrium position on account of viscosity but in an oscillatory manner. As in the earlier case of mechanical forcing, we can derive an expression for the natural frequency if we take the fluids to be a pure conductor in contact with a perfect dielectric. This expression will now take the form of equation (3.1), again derived for a case of perturbed inviscid fluids. This equation which is presented here without a formal derivation will follow later in this article from a more general model where viscosity is entertained. The variables in the new equation are as described for equation (2.1) and the additional variables are DC, the constant voltage, $\epsilon_{0}$ the permittivity of free space (also known as vacuum permittivity), and $\epsilon_{2}$, the relative permittivity of fluid 2, where we take the top fluid which is taken to be an isotropic dielectric as an example. We can immediately see from the signs of the terms that the addition of a constant electric field helps to offset the effect of gravity. The natural frequency in the electrostatic case, now termed $Im(\sigma_{e})$, is given by

3.1 $$\sigma_{e}^{2}=-k\frac{\left(g\Delta \rho -(\epsilon_{2}\epsilon_{0}\;\coth\left(kH_{2}\right)/h_{2}^{2})(DC^{2})k+\gamma k^{2}\right)}{(\rho_{1}+\rho_{2})\coth\left(kH_{2}\right)}.$$

The $DC^{2}$ term arises from the Maxwell stress that appears as a quadratic term (hence the square of DC) in the normal component of the interfacial momentum balance (compared with §3(b)). It is thus possible to impose a voltage to oppose gravity and thereby alter the natural frequency and, more importantly, make the effect of interfacial tension, γ, more prominent. The quadratic nature of DC implies that it does not matter which plate is grounded and which plate is subject to the electrostatic field. While the aforementioned formula, viz., equation (3.1), is derived under the assumption of inviscid fluids, adding viscosity does not, in principle, change the qualitative aspect of the physics. Observe that the k dependence of the DC term in the aforementioned formula is one order higher than the gravity term. This forecasts the possibility that at low wavenumbers, a DC field will cause a Rayleigh–Taylor like behaviour with pillaring of the interface. We shall return to this aspect of electrostatic forcing later in our discussion of electrostatic-induced resonance to which we now turn.

**Figure 11.**
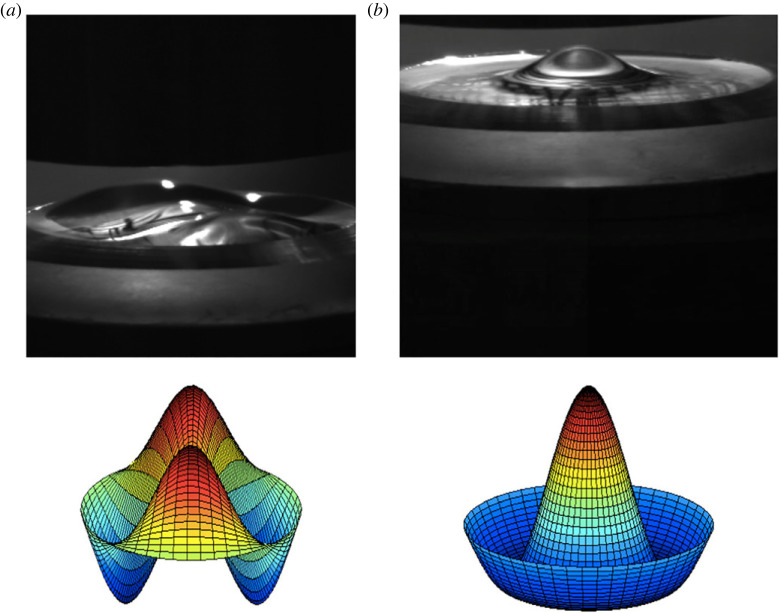
Two photographs of electrostatic resonant waves at water–air interface in a petri dish with a metallic bottom. The modal patterns are (a) (2,1) and (b) (0,1). The photographs are due to K. Ward (private communication) and taken at JAXA, Japan. (Online version in colour.)

As a visual example of electrostatic resonant instability, see figure 11. It depicts observations of the electrostatic instability when a petri dish containing water is subject to an oscillatory electrostatic field. Two modal structures are seen alongside the corresponding mathematical modes that would result from a theory which is described in §3(b). The modes, as in the mechanical forcing case, are indicated by two indices, the first one, m, is the azimuthal variation (which goes as cos(mθ)), while the second index, n, is the radial mode and corresponds to the nth root of $J_{m}^{\prime }(k_{n}R),$ where R is the radius of the cylinder.

In a container that is parametrically forced with a frequency, ω, a critical oscillatory voltage must be reached before resonance is obtained and patterns are seen at the interface. In any analysis, the inputs are the fluid properties, depths, lateral wall geometry and parametric or forcing frequency. The predictions from the calculation for the onset of the instability are the critical amplitude of voltage, the corresponding wavelength or equivalently the wavenumber, and the onset velocity and pressure profiles. We turn to a mathematical model that leads to such predictions.^2^

### (b) The mathematical model—perfect conductor in contact with a perfect dielectric

The mathematical model refers to a three-dimensional description of two incompressible, immiscible, Newtonian fluids of infinite lateral extent, lying between two rigid electrically conducting plates located at $-H_{1}$ and $H_{2}$, across which a voltage difference, A cos(ωt), is applied (compared with figure 10 with D=0) and where there is no constant DC voltage being applied. The bottom fluid is assumed to be a perfect conductor, while the top fluid is taken to be a perfect dielectric. The governing equations use length and time scales given by $H_{1}$ and $t=H_{1}/U$ and a potential scale given by A. Here, U is a characteristic velocity scale. It will be seen that the velocity perturbations at the neutral stability state vanish. The scaled potential field is given by

3.2 $$\;\nabla^{2}\psi =0.$$

The scaled potential field is cos(Ωt) at the bottom plate, i.e. at z=−1, while the top plate at z=H is assumed to be grounded, where $H=H_{2}/H_{1}$. The equations of motion are as follows:

3.3 $$\;\delta_{j}Re\left(\frac{\partial {\text{v}}_{j}}{\partial t}+{\text{v}}_{j}\cdot \nabla {\text{v}}_{j}\right)=-\nabla {\text{p}}_{j}+\eta_{j}\nabla^{2}{\text{v}}_{j}-\delta_{j}G{\text{i}}_{\text{z}},$$

where the index j represents the bottom fluid (1) and top fluid (2). Here, $G=\rho_{1}gH_{1}^{2}/\mu_{1}U$, $Re=\rho_{1}UH_{1}/\mu_{1}$, $\delta_{j}=\rho_{j}/\rho_{1}$ and $\eta_{j}=\mu_{j}/\mu_{1}$. No slip and no penetration boundary conditions are imposed on the rigid walls at z=−1 and z=H. The common interface is located at z=ζ(x,t). The velocity components at the interface are equal, and the normal and tangential components of the momentum balance hold, i.e.

3.4 $$\;{\text{v}}_{1}\cdot \text{n}=U\;\text{and}\;{\text{v}}_{1}={\text{v}}_{2}$$

and

3.5 $$\;[[-{\text{p}}_{j}\text{I}\cdot \text{n}]]+[[\eta_{j}{\text{T}}^{H}\cdot \text{n}]]-({\text{T}}^{M}\cdot \text{n})=-\frac{1}{Ca}\text{n}\nabla \cdot \text{n},\;\text{where}\;Ca=\frac{\mu_{1}U}{\gamma }.$$

Here, the double brackets $\left[[\phi_{j}]\right]$ represent $\phi_{1}-\phi_{2}$, I is the identity tensor and the interfacial speed, U, the unit normal vector, n, and the unit tangent vector, t, are given by

3.6 $$\;U=\frac{\partial \zeta /\partial t}{[1+(\partial \zeta /\partial x{)}^{2}{]}^{1/2}},\;\text{n}=\frac{-(\partial \zeta /\partial x)i_{x}+i_{z}}{[1+(\partial \zeta /\partial x{)}^{2}{]}^{1/2}}\;\text{and}\;\text{t}=\frac{i_{x}+(\partial \zeta /\partial x)i_{z}}{[1+(\partial \zeta /\partial x{)}^{2}{]}^{1/2}}.$$

In equation (3.5), the hydrodynamic stress tensor, i.e. $T^{H}$, takes its usual form, i.e. $\nabla v_{j}+{\left(\nabla v_{j}\right)}^{t}$ and the dimensional form of the Maxwell stress tensor, i.e.$T^{M}$, (compared with [22]), is given by $T^{M}=\epsilon \epsilon_{0}EE-\frac{1}{2}\epsilon \epsilon_{0}E\cdot EI$, where $E=-\nabla \psi_{2}$ is the electric field, ϵ is the relative permittivity of the fluid and $\epsilon_{0}$ is the permittivity of free space. The normal component of the Maxwell stress in dimensionless form can be written as follows:

3.7 $$\;({\text{T}}^{M}\cdot \text{n})\cdot \text{n}=\frac{D}{2}[{\left(\nabla \psi_{2}\cdot \text{n}\right)}^{2}-{\left(\nabla \psi_{2}\cdot \text{t}\right)}^{2}],$$

where $D=\epsilon \epsilon_{0}A^{2}/H_{1}\mu_{1}U$. For the case of a perfect conductor-dielectric model, there are no tangential components of the Maxwell stress tensor that make a contribution (compared with [22]).

Four key dimensionless groups, i.e. Re, Ca, G and D, evolve from the non-dimensional governing equations and boundary conditions (3.2)–(3.7). In what follows, it will become evident that of these groups, GCa and DCa will combine to form two principal dimensionless groups on account of the base state being quiescent. Our aim is to determine the stability of this simple equilibrium base state.

In the case of inviscid fluids and for equal fluid depths, i.e. H=1, the aforementioned model simplifies immensely. The principal equations are $\nabla^{2}p_{j}=0$ subject to Neumann conditions at the plates and the first of equations (3.4) and (3.5) excluding the viscous term. This special case of resonance in inviscid fluids was studied by Yih [23] and Briskman and Shaidurov [24] (almost simultaneously!). Here too, as in the case of mechanical forcing, a Mathieu equation is obtained. It takes the form

3.8 $$\frac{\partial^{2}\zeta^{\prime }}{\partial t^{2}}+[p-2q\;cos(2\Omega t)]\zeta^{\prime }=0,$$

where p and q are given by

3.9 $$p=\frac{\left(1-\delta )G\;Ca\;k+k^{3}-(1/2)DCa\;k^{2}\;\coth(k\right)}{Re\;Ca(\delta +1)\;\coth(k)}\;\text{and}\;q=\frac{D\;Cak^{2}}{4\;Re\;Ca\;(\delta +1)}.$$

**Figure 12.**
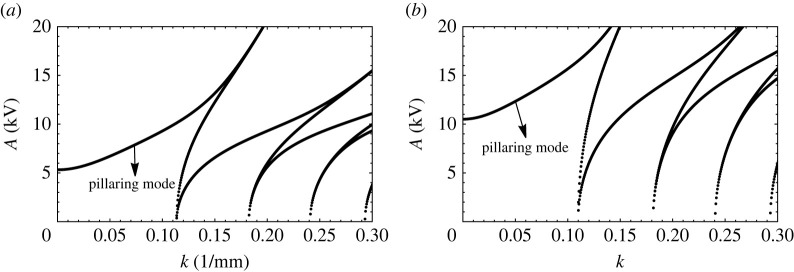
Critical A versus k for a bilayer of water/silicone oil for an applied frequency of 2 Hz and thicknesses of (*a*) $H_{1}=H_{2}=1.27\;\mathrm{cm}$ and (*b*) $H_{1}=H_{2}=2.54\;\mathrm{cm}$. Properties are given in table 4.

**Figure 13.**
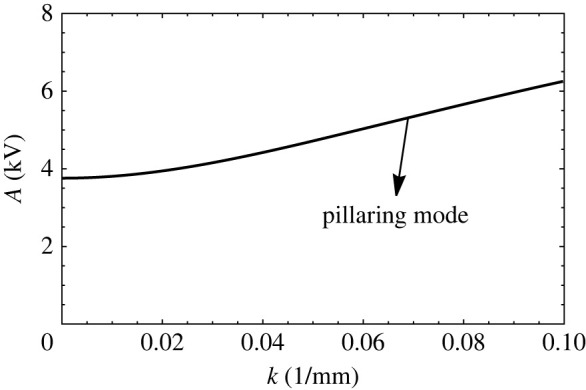
The critical A versus k plot obtained for a bilayer of water and silicone oil of thicknesses $H_{1}=H_{2}=1.27\;\mathrm{cm}$ in the absence of electrostatic resonance and in the presence of constant potential. Other properties of the fluids are given in table 4.

**Figure 14.**
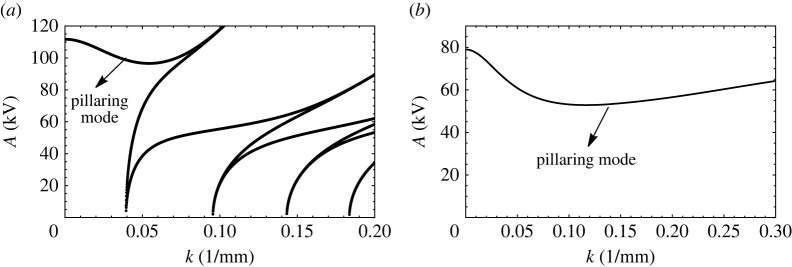
The critical A versus k plot obtained for a bilayer of water and air of thicknesses (*a*) $H_{1}=H_{2}=3.8\;\mathrm{cm}$ and in the presence of AC electrostatic forcing of frequency 2 Hz (*b*) in the presence of only DC electrostatic forcing [25]. The physical properties of the fluids are given in table 4.

A plot of ‘A’ vs k follows upon evaluation of p vs q in the aforementioned Mathieu equation for a given Ω, and to be specific, we use the physical properties entered in table 4. The curves take the shape as depicted in figures 12*a*,*b*, where we make two observations. First, there appears a portion of the plot preceding the usual tongues, and second, this region is substantially raised when the fluid heights are increased. The origin of this preliminary region marked in figures 12*a* is the $\frac{D}{2}$ term in p. This is so we can simply plot A vs k when oscillatory forcing is eliminated and replaced by a pure DC field. Then, figure 13 results. The curve in this figure is a consequence of the competition between a destabilizing DC field that counteracts the stabilizing gravitational field and surface tension. For large k and low GCa, the problem acts like a form of the Rayleigh Taylor instability, where now the destabilizing DC field competes with surface tension. This leads to an ostensibly monotonic increasing curve. This conclusion, however, is misleading and is seen when GCa is increased substantially. To see this, we turn to figure 14*a*, which is drawn for a water/air system that has a low value of density ratio, δ and a high GCa, i.e. a high Bond number. Observe the non-monotonic nature of the region ahead of the first tongue. The dip or minimum point in the curve is indicative of a competition in wavenumber. Indeed at low wavenumbers, the competition is between gravity and the $\frac{D}{2}$ term, which is a pseudo DC field, whereas for high wavenumber, the competition is between the electrostatic field and surface tension. This dual role of the wavenumber leads to the ‘dip’. This is better exemplified by figure 14*b*, drawn for a pure DC field [25]. This region of wavenumbers in both figures which we have just described is often called the ‘pillaring’ mode and marked as such as it is in this region that pillars of fluid are formed, leading to an electric shorting of the electrodes [26].

**Figure 15.**
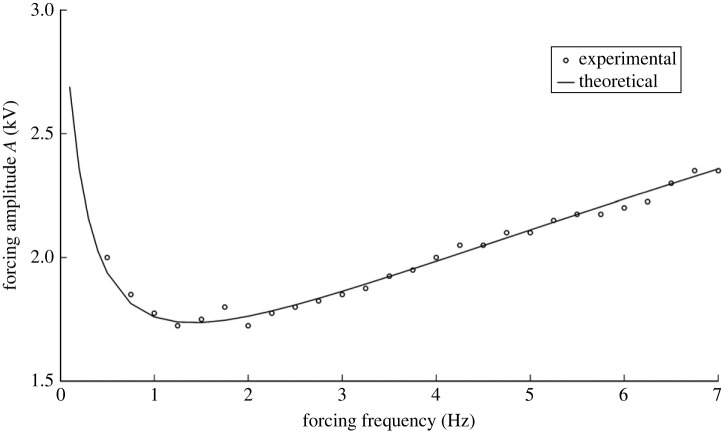
Comparison of theoretical prediction with experiment of onset voltage vs frequency in a silicone oil-water system. Physical properties are given in table 4. The depths of the fluid layers are $H_{1}=0.033\;m$ and $H_{2}=0.005\;m$. The radius of the Faraday cell is 0.0635 m. See Ward *et al.* [26]. Reprinted with permission from Cambridge University Press.

**Table 4.** Fluid properties for water, silicone oil and air. These fluids are chosen due to the nature of their electrical properties, which allows for Faraday instability thresholds that are obtainable with realistic voltage drops.

physical properties	water	silicone oil	air
density ρ (${\mathrm{kg}\;m}^{-3}$)	1000	856	1.225
kinematic viscosity ν ($m^{2}\;s^{-1}$)	$10^{-6}$	$1.5\times 10^{-6}$	$15.52\times 10^{-6}$
relative permittivity ϵ	80	2.36	1.0059
conductivity σ (${S\;m}^{-1}$)	$5.5\times 10^{-6}$	$2\times 10^{-13}$	
interfacial tension γ (${N\;m}^{-1}$)	0.045		0.07275

### (c) Some experimental comparisons—the case of viscous fluids

In practical situations, the fluids are not inviscid and in many cases nor is the fluid pair a perfect dielectric/conductor. Taking viscosity and charge accumulation on the interface between fluids into account is important. To accommodate this feature in the physical description of the instability, a leaky dielectric model has been used (compared with Taylor [27] and Melcher and Taylor [28]). The derivation of the leaky dielectric model is reviewed extensively and applied to several problems in the 1997 survey article by Saville [22]. In brief, the model is composed of the equations of motions coupled with electromechanical stresses at the fluid interfaces resulting from an accumulation of charge, allowing for departure from the stresses that occur in perfect conductors or dielectrics. The leaky dielectric model with periodic forcing has been studied with a long wave or lubrication approximation by Gambhire and Thaokar [29] and later applied by Roberts and Kumar [30,31] to investigate the control over pillaring instabilities in thin polymer films. In both of these studies, fluid inertia was ignored as the objectives were not to study Faraday resonance. Rather, they were meant to investigate how periodic forcing could stave off pillaring patterns otherwise observed in leaky dielectric thin films under DC fields.

Given the properties in table 4, it is apparent that for experiments that use such fluid pairs, a perfect conductor/perfect dielectric model may be used [26,32]. An experiment by Ward et al. using a wide circular container of fluids led to a favourable comparison with theoretical predictions of critical forcing amplitude vs applied frequency. This is depicted in figure 15. The main purpose of the experiments was to show that the data could be used to estimate surface tension by curve fitting models with different assumed surface tensions to the experimental data. A significant improvement of the experimental apparatus, now verifying the predicted wavelengths was produced by Dehe *et al.* [33,34].

**Figure 16.**
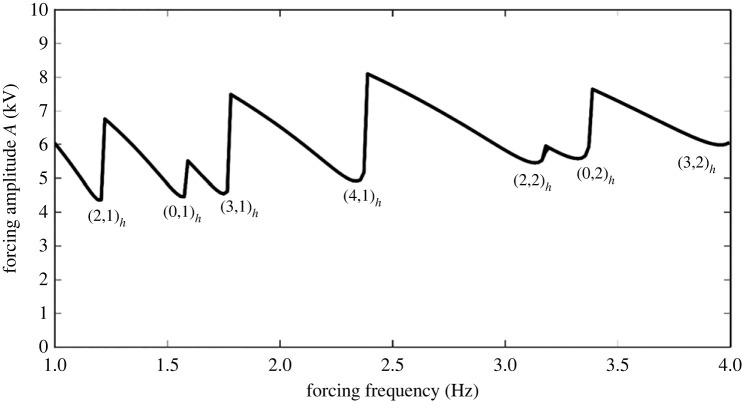
Forcing amplitude A (kV) versus forcing frequency in a bilayer of water and 10 centistoke silicone oil. Each has radius of 25.4 mm and depths of 12.7 mm. Physical properties are given in table 4, where now the kinematic viscosity is $10\times 10^{-6}\;m^{2}\;s^{-1}$ and the density is $935\;{\mathrm{kg}\;m}^{-3}$.

**Figure 17.**
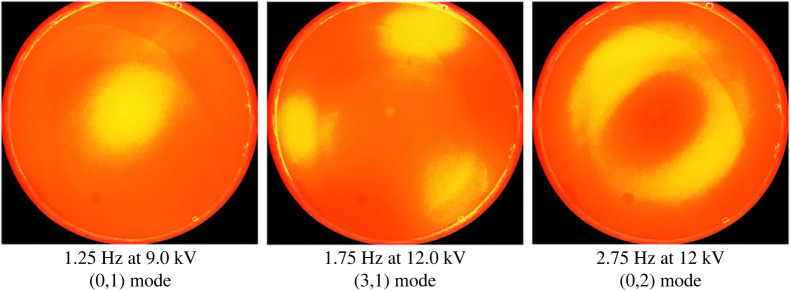
Experiments of electrostatic Faraday forcing of a bilayer of silicone oil and water—unpublished experiments using a transparent electrode done by co-author J. Livesay and co-workers at the University of Florida. The fluid properties are given in table 4. The fluid depths are 12.4 mm each, and the cell radius is 25.4 mm. (Online version in colour.)

To see how mode discretization occurs in a manner similar to mechanical forcing, we may produce a curve of A vs frequency of forcing upon choosing a container geometry and its lateral dimensions. The case of a cylindrical container for the silicone oil/water system is computed and depicted in figure 16. The experimental depictions for the same dimensions are shown in figure 17. Observe that the modes depicted in the figure agree well with the predictions with the exception of the mode at 2.75 Hz, where the pattern could be strongly influenced by axisymmetric mensicus waves. More studies on mode discretization are currently in progress for this type of resonant forcing. In addition to that mentioned earlier, it is observed that all the modes are harmonic with the applied frequency. This comes as no surprise as we do not have a DC offset in the experiments and the Maxwell stress goes as $\cos^{2}(\Omega t)$, which translates into a cos(2Ωt) forcing. A subharmonic response to such forcing is half of the 2Ω and thus harmonic with the actual forcing frequency.

## 4. Some comments on future directions

We close this review with suggestions for future directions based on observations that we find interesting.

The nature of the instability is of interest for both types of resonance. For example, the physical reasons for the occurrence of subcritical motion in the case of mechanical forcing and its apparent absence in electrostatic forcing have not been clearly understood and are deserving of further investigation. A second observation that invites investigation is the sudden transition from one mode to the next with a steep rise in the A vs f curve for electrostatic forcing as opposed to mechanical forcing. A third connected question that is of interest is: What happens at the co-dimension two points in electrostatic instability and do they behave like mechanical resonance? A fourth observation that invites an explanation is that the resonance at low gravity is always subharmonic. Other interesting questions that deserve to be addressed are can we get multiple harmonicities in an experiment and can we overcome mechanical instability with a superposition of electrostatic forcing? Clearly the area is rich with physics, and the questions raised in this area suggest more research.

## 5. Summary

Two main ways to induce resonant instabilities, i.e. mechanical and electrostatic, are reviewed with a focus on recent works that emphasize mode discretization and experimental validation of the theory. In the case of both mechanical and electrostatic Faraday instability, inviscid theory can forecast the modal response during resonance in viscous fluids but not the onset conditions. Experiments show excellent validation of the theoretical predictions provided side wall damping is reduced and stress-free conditions on the side walls can be closely approximated. Low gravity experiments in the case of mechanical Faraday show, in agreement with theory, that high spatial frequency is a feature at low parametric temporal frequency. This is due to the enhancement of the interfacial tension effects in the absence of gravity. An unexplained characteristic of mechanical Faraday resonance is the occurrence of subcritical motion.

## Data Availability

This paper does not contain new data other than figure 17 which is unpublished and available upon request from the communicating author.

## Footnotes

1 Observe that the acceleration is perpendicular to the flat interface and that the fluid system is at rest with respect to an observer on the moving frame. Another way to excite an instability is to impose acceleration parallel to the interface. But here, a laterally confined fluid system cannot be at rest in the moving frame. Such an acceleration leads to ‘frozen waves’ instability. It is interesting in its own right, but it is not the focus of the article [2,3].

2 This is the implication mentioned earlier. As the critical amplitude and wavelength are determined by knowing the properties, it follows that a comparison of experimental critical amplitude with the predicted critical amplitude will yield the interfacial tension, provided the critical amplitude is, in fact, sensitive to the interfacial tension. This implies that the natural frequency needs to be sensitive to the interfacial tension. We believe that this can be achieved in the case of electrostatic forcing by suitably adjusting the DC voltage, whereas it is difficult to do so in the mechanical Faraday forcing without going to microgravity.
